# Safeguarding the best interests of children of incarcerated mothers

**DOI:** 10.1007/s00787-020-01664-8

**Published:** 2020-10-31

**Authors:** Jörg M. Fegert, Liliane Kistler Fegert

**Affiliations:** 1 Head of Department of Child and Adolescent Psychiatry, Psychotherapy, University of Ulm, Ulm, Germany; 2 Head of Penal and Law Enforcement Authority Kanton Schwyz, Schwyz, Switzerland

This letter is a response to Ref. [1]. We agree with the authors that mother–child attachment and negative consequences of incarceration for the mother–child-relationship merit more attention of child and adolescent psychiatrists and society. We agree that a separation at a very early age might cause developmental and attachment problems. Children of incarcerated parents are at risk for short-term and long-term mental health problems. 

Ref. [2] estimates that 50% of US prisoners have children under the age of 18 years. Only a minority of incarcerated parents are female. In 2013, we conducted a survey in the German State of Baden-Württemberg [3]. In a representative sample of 1.551 prisoners, we identified 487 incarcerated persons (31.4%) with children under the age of 18 years. A quarter, corresponding to 147 children, was in the age range 0–3, another 30% in the age range 4–8 years. In this sample, we only identified 32 incarcerated mothers (6.6%). In contrast to the practice of rigid separation at the child’s age of 18 months for example in France and many other countries, in Germany or Switzerland children of incarcerated mothers generally stay with their mothers in special conditions of imprisonment until pre-school or school age. Legally feasible non-custodial settings like the use of electronic monitoring are preferred over imprisonment in the best interests of the child. In an attachment-based parent–child project called “CHANCE”, we supported [4] a subgroup of mothers. Usually, children went to daycare at the prison, while their mothers went to work. The rest of the time, mothers and children spent their time together in a setting comparable to youth welfare mother–child institutions.

In line with some earlier texts from a feminist and human rights perspective, the authors blame society for the individual consequences of detention [5, 6]. Golden (Page 56) even speaks of a “public assault on children”. In line with this argument, Ref. [1] stated that societies choose to separate children from their mothers, only because they consider a mother dangerous to society.

Incarcerated mothers can be dangerous to their children and society. Therefore, an individual forensic risk assessment is necessary in the best interests of the child and the interests of society. Mothers with a personality disorder might maltreat their children even in prison. There are female sex offenders that might sexually abuse their children or radicalized Jihadist mothers might think about sacrificing their children in Jihad. From a developmental perspective, the basic needs of young children can be met in an adapted penal setting, because infants, toddlers, and pre-school children need a close and nurturing relationship with a primary caregiver. However, if the mother–child relationship is troubled by severe psychiatric problems, a separation from the incarcerated mother and placement of the child in foster care might be in the best interests of the child.

In their contribution, Ref. [1] discussed the situation of mother’s members of IS who had gone to Jihad as members of IS and are now returning to European countries together with their children born during Jihad. Some of these mothers are still radicalized and dangerous to societies. At the same time, a folie-à-deux-like indoctrination of children can become a massive danger to the development. Therefore, in every case a forensic criminal risk assessment and an assessment of the quality of the mother–child relationship and developmental risks and child psychopathology are needed to find a solution that is in the best interests of the child.

The literature on adverse childhood experiences (ACE) shows that children of incarcerated parents often suffer from multiple household dysfunctions such as drug abuse, domestic violence, and child abuse. In a representative sample of the German population [7], 88 participants out of 2.531 (3.5%) reported to have grown up with an incarcerated parent. The association between growing up with an incarcerated household member and depression, anxiety, and compromised general health status was mediated completely by child maltreatment, meaning that the developmental risks of these children can be explained fully by the higher risk for maltreatment and not by the fact of incarceration [7].

We agree that early separation should be avoided. Many incarcerated mothers have own experiences of violence that might put their children at risk of a transgenerational transmission of violence. Therefore, individual assessment is needed. Even a highly dangerous mother that hates our society can stay together with a baby or a toddler in an appropriate context of incarceration if she has a caring relationship to her child. From a children’s rights and developmental needs perspective, adequate and safe settings for imprisonment should be provided.
